# Lived experience of intimate partner violence among women using antiretroviral therapy and other outpatient services in Wolaita Zone, Ethiopia: a phenomenological study

**DOI:** 10.1186/s12978-020-01044-0

**Published:** 2021-02-01

**Authors:** Mengistu Meskele, Nelisiwe Khuzwayo, Myra Taylor

**Affiliations:** 1 School of Nursing and Public Health, Discipline of Public Health, University of KwaZulu-Natal, Durban, South Africa; 2 School of Public Health, Wolaita Sodo University, P.O.Box: 138, Soda, Ethiopia; 3 School of Nursing and Public Health, Discipline of Rural Health, KwaZulu-Natal, Durban, South Africa

**Keywords:** Intimate partner violence, Phenomenology, HIV, Wolaita, Ethiopia

## Abstract

**Background:**

Ethiopia is one of the nations which has an enormous burden of intimate partner violence (IPV), and where it is usually difficult to talk about HIV separately from IPV.

**Objectives:**

This research aimed to explore the lived experience of IPV against women using antiretroviral therapy (ART) and other outpatient services in Wolaita Zone, Ethiopia

**Methods:**

We used an Interpretive (hermeneutic) Phenomenological Analysis design among purposively selected adult women aged 18–49 years. A total of 43 women participated in this study, of whom 30 were using ART, and 13 women were using other health services. We used an in-depth interview and focus group discussions until data saturation, while conscious of the need to maintain the scientific rigor, dependability, and credibility. The data were transcribed verbatim and translated into English. We read the transcripts repeatedly to understand the content. We used NVivo 11 software to assist with data organisation, and also, we used the framework analysis method.

**Results:**

We identified five themes, namely: “women's terrifying experiences of violence,” “the effect of violence on women's health,” “support/lack of support /partner’s controlling behaviours,” “women’s feelings about the available services,” and “IPV prevention strategies from the perspective of women.” Interviewees described their violent experiences which included wife-beating, being stigmatised in front of others, having material thrown at the woman’s face, wife’s hand and teeth were broken, forced sex, restriction of movement, name-calling, threats to hurt, being insulted, being left alone, and the withdrawal of finances. The negative health impacts reported included abortion, infection with HIV and other sexually transmitted diseases, disability, child’s death, and depression. The disclosure of HIV test information resulted in violence. Inappropriate punishment of the perpetrator and the lack of a supportive women’s network to avert IPV were perceived as legal limitations.

**Conclusions:**

IPV is a considerable health burden, varying in its presentation and its negative impact on women’s health. Improved laws should provide justice for all victims. Establishing a women’s network to assist women at risk of violence, should be emphasised. Unwise HIV test result disclosure leads to IPV; hence HIV disclosure should be facilitated through health care providers.

## Plain English summary

Intimate partner violence (IPV) and HIV infection present as an overlapping/ intersecting challenge. There are gender gaps in the justice system in Ethiopia, and to date, IPV and rape cases have been delayed and given low priority.

We interviewed 43 women who were using antiretroviral therapy and/or other health services from the 19 health facilities in Wolaita Zone, Southern Ethiopia. One or two women were picked from each facility purposively until we reached information satiety for the in-depth interviews. We also conducted four focus group discussions with different women.

We found five main themes from this study: “Women’s terrifying experiences of violence,” “The effects of violence on women’s health,” “Support/lack of support /controlling behaviours/,” “Women’s feelings about the available services,” and “IPV prevention strategies from the perspective of women.” Women who were HIV positive, suffered further at the hands of their partners. Alcohol consumption and the use of substances by male partners took a heavy toll on women’s experience of IPV, and the psychosocial consequences of IPV reported were fear, discrimination and divorce.

The available legal system, women’s affairs office, and the roles of women leaders were seen as ineffective. Besides that, the participants believed that there was an absence of a women’s network actively working on IPV. They recommended that health care workers should assist women in the disclosure of HIV test results to their partners. Further, there existed weak and inappropriate punishment for perpetrators of violence.

In conclusion, IPV is a considerable health problem in Wolaita. There is a need to examine the relationship between IPV and HIV in the different parts of the country.

## Background 

Violence Against Women (VAW) takes place in almost all countries, amongst girls and women of all cultures, ages, race, educational level, religions and sexual orientation [1]. Accordingly, in the ever- partnered women, the prevalence of physical or sexual violence or both, by an intimate partner ranged from 15 to 71% [2]. The World Health Organization (WHO) defines VAW as “any act of gender-based violence that leads to or is probably going to end in, sexual, physical, or psychological suffering or harm to women, including threats of such acts, bullying or arbitrary deprivation of liberty, whether occurring publicly or privately in life,” perpetrated by a current or former partner [1].

Globally, intimate partner violence (IPV) is a tremendous public health problem with a global prevalence of 30% for physical and/or sexual IPV among ever-partnered women [3]. Intimate partner violence is associated with HIV infection among women in Africa [4, 5]. Intimate partner violence and HIV are overlapping/intersecting challenges, with a significantly high prevalence among women who are living with HIV/AIDS [4–6].

To protect the rights of women and to promote gender equality and equity, Ethiopia revised its family law in 2000, as well as its criminal code in 2005 [7]. Moreover, Ethiopia is a signatory to the convention on international human rights. However, there are gender gaps in the justice system, insufficient investigations, and a lack of special handling of the cases involving women and children. Furthermore, to date, IPV and rape cases have been delayed and given low priority [8, 9].

In Ethiopia, some women and men tolerate and accept wife-beating, which makes IPV prevention difficult. For instance, 28% of men and 63% of women agreed that a husband is justified in beating his wife [10]. Although the government of Ethiopia is trying to empower women, some traditional and conservative elements in society still need an attitudinal change (both men and women), in their belief that wife-beating is normal, and in the practice of harmful traditions like marital rape, and the idea that women are not equal to men [9].

Currently, according to the 2016 Ethiopian Health and Demographic Survey (EDHS) report, IPV affects the economy and the health, and human rights of women. In Ethiopia, more than one in three of ever-married women (35%) reported that they had experienced physical, emotional, or sexual violence from their husbands or partner at some point in time. One quarter (24%) of women experienced psychological abuse, a quarter (25%) experienced physical violence, while (11%) experienced sexual violence. Most IPV incidents (38%) occurred amongst women in the older age groups (40–49 years) [10, 11]. Earlier studies also revealed that after HIV serostatus disclosure, one in three women experienced partner violence [12, 13]. Some women experienced controlling behaviour by their partner, including emotional abuse, denial of communication, blame, abandonment, refusal to use safer sex methods, withdrawal of marital support, and marriage dissolution, stigma, and violence [12, 13].

Ethiopia is no exception since the occurrence of VAW is also high. The government of Ethiopia has been revising different documents to guarantee the rights of women, and the country has highlighted that further reliable information is needed to protect these rights [11].

Therefore, this study aimed to explore the context and the lived experience of IPV among women who were using ART and other health services in Wolaita Zone, Southern Ethiopia.

## Methods

### Study area and setting

This study was conducted in Wolaita Zone, one of the 14 Zones in the Southern Nations, Nationalities, and People’s Regional State (SNNPR) of Ethiopia. The Wolaita language is the native language of the Zone. Wolaita Sodo, the capital town of Wolaita Zone, is 330 km southwest of Addis Ababa, the capital city of Ethiopia. According to the 2007 Central Statistical Agency of Ethiopia (CSA), a total of 2,473,190 populations are living in the Zone [14]. In Wolaita Zone, we have 19 health facilities which were providing ART, from which we randomly selected nine health facilities.

### Study design

We used an Interpretative (hermeneutic) Phenomenological Analysis (IPA) design to explore the lived experiences of women and to document the participants’ lives [15]. We chose this method because it is very good at bringing out what is usually hidden in human experience and human relations. The IPA design requires the interpretation of the narratives provided by participants, and the expert knowledge on the part of the researcher is valuable. The IPA design can go beyond the descriptive, in looking for the meaning hidden in standard life practices [16, 17]. The use of IPA helped us understand how individuals make sense of their IPV experience [18]. The study participants were actively engaged in interpreting the events, objects, and people in their lives.

Moreover, we tried to understand our participants’ perspectives of IPV to make the analysis more productive and more comprehensive [18]. We undertook the in-depth interviews and Focus Group Discussions (FGDs) with women until we achieved data saturation. These assisted in the understanding of their personal experiences, and several individuals shared such experiences [18]. These focused on what the participants have in common concerning their IPV experiences, the description of such events and how it affects their lives. The different meanings that women attach to their experiences were studied. The research questions were “What have the women who were using ART, and family planning, prevention of mother to child transmission (PMTCT), and antenatal care (ANC) experienced concerning IPV and what contexts have influenced their experience of IPV” [19].

### Selection of study sample

We selected the participants for the in-depth interviews and focus group discussions (FGDs) from the 19 health facilities which were providing ART. One or two women were picked from each facility purposively until idea saturation was reached for the in-depth interviews. Moreover, for the four FGDs, each FGD included eight to 12 participants who were also selected purposively. Three of the FGDs were with women who were using ART, while one of the FGDs was with HIV negative women who were using ANC and family planning services. The health care providers assisted in the selection of the study participants to obtain a rich explanation about the phenomenon.

We also included peer educators and adherence counselor supporter women who were using antiretroviral therapy (ART) in our FGDs. In total, we conducted 13 in-depth interviews and four FGDs among women who were using antiretroviral therapy (ART) and family planning, ANC, and PMTCT. A total of forty-three women participated in this study, of whom 30 were using ART, and 13 women were using other health services.

### Data collection and procedures

We conducted the data collection from October to November 2018. The facilitator guides were adapted from the World Health Organization’s (WHO) practical guide on researching violence against women [20] (Additional file 1: S1_file). The principal investigator (PI) conducted face to face in-depth interviews and facilitated the four FGDs. During the meetings and discussions, where necessary, the PI probed to get further clarity from the participants. Four experienced female research assistants (RAs), who were able to speak the local language Wolaita and the Amharic language fluently, assisted the PI with note taking and recordings during the FGDs. We conducted all in-depth interviews at offices in the health facility or the participants’ preferred places to maintain privacy and confidentiality.

The PI initiated “warm-up” discussions to reduce the tension among the participants. After we had examined, analysed, and understood the first interviewee women’s IPV experience in detail, we then moved to an equally attentive exploration of subsequent interviews until data saturation [18]. We purposively selected eight to 12 women for each FGD. We scheduled meetings based on women's preferences. The number of interviews was determined by achieving saturation of the ideas. We also provided refreshments for the FGD participants. We used the scientific rigor and integrity [21] to maintain trustworthiness of the study.

### Data analysis

We used a framework analysis method, which followed seven steps. These were transcription, familiarisation with the interview, coding, developing a working framework, applying an analytical framework, charting data into the framework matrix, and interpreting the data [22, 23]. In the first transcription stage, the data were audio-recorded and transcribed verbatim and translated into the English language by the principal investigator and research assistants. In the second familiarisation phase, the authors read and re-read the transcripts several times. This stage was followed by listening to the audio recordings a few times to get new insights. Also, we took notes and considered our observations and reflected on the interview experiences. The initial interpretive comments made were identified and recorded. Thirdly, we identified codes, which we produced from reading the texts of the transcripts. We used NVIVO 11 software to assist with the data organisation and analysis. Fourthly, we developed a working framework, in which we compared the labels and agreed on the set of codes to apply in the subsequent transcripts. Then the codes were grouped into categories. The process formed the working analytical framework. In the fifth step, we performed the analytical framework by indexing the succeeding transcripts using the available codes and categories. In the sixth step, we charted the data into the framework matrix, using a spreadsheet. It involved summarising the data by groups from the transcripts of the study. The final and seventh step was interpreting the data, which began with writing a narrative account of the study. Then themes were derived from the data. It also clarified each of the identified themes by describing them, and examples were given from interview(s), followed by analytical comments from the researchers.

## Results

### Socio-demographic characteristics of the study population

Of the total of 43 women, aged between 20 and 45 years, thirty of the participants were living with HIV. Over half of the participants were married, 25 (58.1%), but 9 (20.9%) were widowed, 6 (14%) were divorced, and the remaining 3 (6.9%) were separated. Most of the participants lived in urban areas, 90.6% (39 out of 43). Almost a fifth of the participants, 9 (18.6%), were not educated (Additional file 2: S2_file).

From the analysis, the following five themes emerged. “Women’s terrifying experiences of violence, “The effects of violence on their health,”; “Support/lack of support /controlling behaviours/,” “Women’s feelings about the available services,” and “IPV prevention strategies from the perspective of women.” The first two themes explain the women’s terrifying experience of physical, sexual, and psychological violence and the multiple health effects on the women, as reported below (Additional file 3: S3_file, Additional file 4).

### Women's terrifying experiences of violence

#### Types of violence

Women's terrifying experiences of violence is a major theme of this study. The data revealed that most of the participants had experienced mixed or overlapping types of terrifying abuse from their intimate partners. The interviewees reported recurrent physical and emotional abuse, but relatively few participants reported sexual abuse. According to the participants, they were beaten with sticks, had material thrown at their faces, and some even had their hands and teeth broken.

Additionally, some indicated that their partners had extramarital relationships, but male partners restricted women in socialising and having contact with other people. As reported by the participants, their extramarital relationships led some women to the acquisition of HIV. The behaviour of their intimate partners included repeated name-calling, threats to hurt, hostility, withdrawal of finances, and forced sex. Women in rural and urban areas shared the same experiences. Both younger and older women reported terrifying experiences of violence by an intimate partner; however, HIV positive status exacerbated IPV.

One of the in-depth interview participants explained:

***“My husband was an alcoholic, and he was older than me. He used to stab me with a knife. One day I stayed the whole night with a knife in my body because no one can take it out of my body. He used to punch me in my face, and my teeth also changed their normal place. Here, the scars in my body that you see emerged because of the bleeding after he had beaten me. He used to drag me on the ground by my hair, and my hair disappeared in his hands two times. Also, my bone in the chest broke after he had beaten me.” (Woman living with HIV, O#2, aged 32).***

A discussant from one of the focus group discussions also explained:

*“In public, when I am around my family and extended family, he belittles and insults me because of my HIV status, he says, you can’t take care of your children, you are going to die because you have AIDS,” He discloses my HIV status to everybody including my friends.” (Participant S1#6, woman aged 37).*

The discussant with HIV discordant result explained:

*“We tested for HIV, and my results come back positive, but my husband was not. He decided to divorce; and torched me psychologically, mentioning, “You are living with HIV infection” Now I do not want to tell all the insults he heaped on me.” (Participant S2#3, woman aged 40).*

There were concerns in the interviews about the severity of the violence and the threats commonly used by their partners to inculcate fear, with the partner threatening to kill or slaughter them. As in the example described above, women who were HIV positive suffered further at the hands of their partners, who stigmatised and belittled them in front of other people. Alcohol consumption and the use of other substances such as chewing “chat” and addiction to “shisha” (a way of smoking tobacco, mixed with molasses, sugar or fruit,) through a tube) by male partners, took a heavy toll on women’s experience of IPV. A discordant HIV result, the extramarital sexual contact of their partners, and women themselves having an affair with another man increased their husband's aggressive behaviour, as did having children from another husband.

### The effects of violence on women’s health

The violence affected both women’s physical and mental health. For instance, the physical effects reported could be both short and long term, as a result of abuse at the individual and social / community level. Participants described many health problems such as miscarriage, acquisition of STI/HIV infections, uterovaginal prolapse, the sequellae of pain around the ear and back, disability, and uterine infections. As two of the women explained, abortion and child death happened as a result of their male partners’ physical abuse when the two partners were quarreling. A few of the interviewees also reported the effects of violence and the resulting disability, which then hindered women from their activities demanding physical energy. Women also reported the negative impact of violence on their daily social engagement with others. For instance, according to the discussants, as a result of the discrimination from their community, women experienced loneliness, fear, and depression, and as a result of divorce, property loss, and the inability to remarry afterward, which were severe effects of violence.

One of the FGD discussants explained her experience:

*“I lost one of my eyes and became blind. I didn't get medical help. I don’t have one of my organs, but I feel good because I am still alive. He was not giving me financial support all my life.” (Participant S2#3, woman aged 40).*

Another of the participants reported:

*“He divorced his wife after three months of marriage. He infected her with HIV/STI. He had multiple sexual partners. He was abusing them sexually. One of his wives had a miscarriage as a result of his beatings. He kicked his wife while she was three months pregnant, and unfortunately, she had a miscarriage.” He was drinking alcohol, chewing “khat,” and smoking (Participant T#2, woman aged 28).*

The FGD discussant described her experience:

***“After two months of giving birth, when we were quarreling, our son fell and died. My husband threatened me if I disclose this issue to other people; “I am going to cut your neck, he said.” I left his home, and I went to my mother's house.” (Participant S1#1, woman aged 38).***

According to the reports from the interviewees, violence not only hurts women’s and children’s health, but it also has an effect on their economy as well as their social and psychological wellbeing. Moreover, the violence affected women’s social status and resulted in their experiencing emotional turmoil. Its effects further resulted in men withholding finances from their female partners.

### Support/lack of support/ women receive from the partner (controlling behaviour of partners)

According to most of the participants, the controlling behaviour of their male partners denied them any possible support. Data from the interviewees indicated that older women were less likely to experienced controlling behaviour than younger women. Women also reported that their husbands closed/locked the door so that they were left in the house, but were not allowed to leave the house, which disturbed their lives. Participants also indicated that men prevented their women from going outside their home, and they even limited their contact to only communicating through the windows.

Moreover, they reported that their husbands/partners also restricted their speech. Additionally, participants received repeated phone calls from their partners who threatened the women, as they were suspicious that their wives were having sex with other men. For, example, one of the interviewees explained this:

***“No person could enter my home. Even if I became ill, no friends were allowed to ask me. When my husband was going to the office, he used to lock the door, and no friend can access and suppose a person is in the home. I couldn’t make a call to contact my friends because I had no phone.”(Participant S1#7, women aged 37).***

Other discussants also explained the condition as:

***“He was restricting my movements. When I was out of my home, he usually makes repeated calls and threatened me as he already knows the place where I was and accuses me of also having sex with other men. My husband doesn't trust me. I will never forget how he violated me when I was in labor.” (Participant S1#9, women aged 40).***

Further, another FDG discussant explained:

*“He closed and locked the door when I was four months pregnant, I spoke through the window, and he severely beat me over and again, asking me why I was hanging out with my friends without his consent. I became dizzy and fell.” (Participant S1#1, women aged 38).*

These actions hindering women from accessing support from their friends, relatives, and neighbors, as described above, and made it difficult for women to access the available support. Moreover, women expressed their psychological trauma, as men were disturbing their lives, insulting them, and preventing them from leaving their own homes.

### Women’s feeling about the available services

The majority of the participants were worried about the lack of implementation of the possible legal service, its inappropriateness, and the weak punishment (which they reported as only 2–3 days’ imprisonment), for the perpetrators. The available legal services are similar in Wolaita Zone, which the government provides to its people. As the participants pointed out, the religious leaders and elders of the communities are also involved in resolving the conflicts among couples. A few participants, however, noted the absence of a legal service to which to report their abuse. Many participants also believed that women lacked awareness about how to use the existing legal service. Some women had sought help from the justice system, but this did not appear to be a typical response. According to the participants, women who live in urban areas and who were educated, were able to defend themselves and received the legal service.

An interviewee also reported that there was no women’s network, effectively working to support women experiencing gender-based violence. However, the majority of women described the availability of other women’s organisations (these comprised one leader to five members and extended to one leader for each of 30 members). One leader to five members is the subset of the one leader to 30 women division aiming to reach women at the grassroots level. In Ethiopia, these organisations were established to accomplish certain governmental activities like infectious disease prevention, vaccination programs, and specific agricultural and educational duties. However, the majority of the women felt that the existing one leader to five members, does not play a functional role in the prevention of IPV and lacks the ability to take strong actions against the perpetrators. Likewise, some women considered that there are no influential women leaders or organisations explicitly working on violence prevention in their community. Moreover, they believed that the available governmental women's affairs’ office was not functioning very well. Women felt that such an organisation was not cdoing its duties correctly and was reluctant to punish perpetrators which was not satisfactory. For example, one participant explained this as:

*“The justice office has only punished the perpetrator with three to four days in prison. The government’s justice is nothing. Though the police took them to jail, the perpetrator could not stay a night in jail; rather, the police were releasing the attacker from jail very soon. The punishment is loose.” (Participant T#4, woman aged 30).*

One of the FGD discussants explained it as:

*“There are no strong and organised women leaders (1 leader for five members), but they were supposed to be resolving some conflicts between women and men. The women44′s leader is not functioning, although they are supposed to be in place to do the job.” (Women, code = S#1, aged 38).*

Another discussant explained it as:

*“The governmental women’s affairs office is a symbol. It cannot give us a solution. They hear our problem, and then they let us write an application letter for our case and to submit it to the court. They do not solve our problems; rather, they let us go back to the community elders for mitigation.” (Participant T#1 and T2, women aged 32 &28 respectively).*

*According to the participants, the religious leaders have an essential role in resolving the violence. However, a few women emphasised that it is difficult to get support from the religious leaders and that they are inaccessible to women who need to report the abuse.*

There were also contrary views from other participants who disagreed with this view stating that:

*“The religious leaders are also concerned with violence, and they follow the case if women report it to them. They also have a good role and sacrifice their time to resolve the issues. The religious bodies are helpful." (Participant S2#5, woman aged 40).*

Most of the participants reached consensus about the available legal system, women’s affairs office, and women leaders’ role and perceived these as too weak to safeguard women from violence. Besides, the participants believed that there was an absence of a women’s network actively working on violence in Wolaita Zone.

### Intimate partner violence prevention strategy in the perspective of women

Participants mentioned many possible IPV preventions strategies. Most of the participants reported that after their HIV test result disclosure, their partners abuse them, and this needs a proper prevention strategy. Some of the participants believed that the right time to disclose an HIV test result, to prevent a sudden violent reaction from their partners, should be when they are pregnant, sick, and at night time when they are about to go to bed. The majority of the participants pointed out that the health care provider should offer HIV testing to both partners at a time when the couples are together. Most of the women believed that disclosure of an HIV test result to their partner was beneficial in that they were able to receive care and treatment. Nevertheless, unwise disclosures (sudden and unplanned revelation, not assisted by health care providers) had resulted in different types of violence.

The discussants also explained that if women knew their positive HIV status before their partners, health care workers should test them again as a new case along with their male partners, and assist the disclosure.

Women believed health care providers had a role as did other women who had some awareness about violence, and that they should teach each other how to obtain legal services. Participants also felt that women should know how to generate their own income. Moreover, a few participants also reported that women should discuss their violent situation with their neighbors, to get advice and support from them.

One of the discussants explained the preferred approach of HIV counselors as:

*“But while she is going to disclose her status, she should be counseled by the health care providers and bring her husband to the health facility, and she should be tested again as a new client together with her husband to convince him, then the health professional should counsel her husband very well. Otherwise, in case if she discloses her issue carelessly, the husband can hurt/abuse her by assuming that it was his wife who infected him with HIV.”(Discussant S1#8, women aged 30).*

The participants described the need for IPV prevention strategies since women are dependent on their husbands.:

*“Women are economically dependent upon their husbands. When women accuse their husbands, they cannot pay home rent; they cannot raise their children because husbands immediately leave them alone.” (Participant S1#9, women aged 40).*

Other participants also mentioned that women who were already affected by abuse should teach others how to handle the situation in an organised manner.

*“It is better if the women, who were already affected by abuse, teach others in an organised manner. It is better if the government also takes strong action upon perpetrators. It is also nice if a specific association of women is working on this issue.”(Discussant S1#8, woman aged 30).*

Some women mentioned that reporting the issue to public prosecutors or the women's affairs’ office or the justice office is crucial. As a solution, women indicated that there is a need to establish a strong women’s network, with members of such a network individually working against violence. Moreover, participants highlighted that the available “one leader to five members and one leader to thirty members” of the women’s army and women’s affairs’ offices of government, has to emphasise IPV prevention activities. Most women agreed that the available laws should be stringent and provide justice for all the victims.

One of the participants explained it as:

*“The one leader to five member’s organisation should work strongly because the women are hiding their secret of abuse. Then the one leader to five members association should wisely ask the abuse experience of women and come up with a possible solution. Also, the woreda women’s affairs should work hard to help women.”(Participant T#1, women aged 32).*

Other participants had further suggestions; for example, one interviewee said:

*“In my opinion, it is better if you gather men and teach them in a group to prevent their abusive behaviours against women. Education is essential for men. It is also better if women and men get education together in violence issues.” (Participant S1#7, women aged 38).*

The discussants were interested in and pointed out IPV prevention strategies that needed to be implemented or implemented more effectively, including wise disclosure of HIV, teaching others in the community, establishing a strong women’s network, teaching male partners together with females, and that the available law should be stringent and provide justice to all victims.

## Discussion

Our study confirms the results of the studies conducted in Ethiopia, such as the Demographic and Health Survey (EDHS) conducted in 2016, and South African studies (11, 24,25). Participants reported similar experiences of IPV, including physical, sexual, and psychological violence. However, the findings of our study further showed that HIV positive women were more vulnerable to IPV than their counterparts. Furthermore, women from rural areas reported many cases of traumatic violence.

We found that IPV in Woloita district was extensive, and the analysis of the results of our study identified and documented women’s terrifying experiences of IPV, including physical, sexual, and psychological violence. Most of the time, such violence was experienced as mixed or overlapping types of violence. Our study further identified that the women reported recurrent physical and emotional violence, but relatively few participants reported sexual abuse. Such violence is not a new occurrence in Ethiopia [11, 24, 25], and it affects both women living with and without HIV. Our research found that the bullying behaviours of the husbands/ partners wear down the women’s self-esteem, and the consistent pattern of abusive words undermines their mental health. A study in South Africa showed that emotional violence was the most prevalent form of abuse reported [24], and the qualitative research in Kenya also reported women’s terrifying experience of IPV in that country [12], indicating the need for further work to address this significant problem in many African countries. Researchers in the WHO study agree that between 10 and 69% of women reported physical abuse, while between 6 and 47% of adult women worldwide reported sexual violence [26]. The overall world estimate of physical and / sexual violence experienced by an intimate partner was that one in three women (30%) had been affected [26].

Previous studies conducted on HIV and IPV in countries, including Kenya [12] and Swaziland [27], show that HIV disclosure of sero-status increased the risk of stigma and withdrawal of financial support to the affected women. The finding that emerged from our analysis indicates that an HIV discordant result and unwise disclosure of an HIV test result to the male partner, also lead women to IPV and stigma. Therefore, it is sensible that women who are planning to disclose their HIV status to their partners must use caution while they are revealing their HIV status, to avoid being physically abused by their partner. Our study participants also emphasised that health care providers should assist in the disclosure of the women’s HIV results to prevent abuse from their partners. These current study findings were also confirmed by previous studies [13, 28].

We report the negative effects of violence on the women’s health, and that they had reported many adverse health effects arising from IPV. Consumption of too much alcohol was an important factor exacerbating the women’s IPV experience. Other substance use, complementing the alcohol consumption that emerged from our study included the use of ‘khat’ (from a tree, *Catha edulis*, which is a stimulant and causes excitement and euphoria) and ‘shisha’ addiction, (which is a way of smoking tobacco mixed with molasses sugar or fruit, through a tube) by male partners. This result supports the finding observed in previous studies by WHO, South Africa, South Western Uganda, and the Center for HIV law policy study [6, 7, 24, 29–31]. Other earlier studies also revealed that men who are alcoholics quickly become aggressive, which contributes to IPV [32]. The physical violence that occurred during the fighting or quarreling among couples can lead to severe health impacts like abortion, child injury, and death. The previous WHO study of global and regional estimates on IPV confirmed our results [6]. Earlier studies conducted in Togo [33] and South Africa [34] show that IPV has resulted in depression. In this study, the women emphasised the physical suffering to which they were exposed through IPV, but IPV also had severe adverse effects on women’s mental health. Women who live with HIV reported it most frequently. Victimisation by an intimate partner has been shown to lead to mental health problems, including anxiety, depression, and attempted suicide [35, 36]. However, this study did not include the harmful effects of IPV on children in the household who grow up in such a milieu [37].

Earlier studies in South Africa and Zimbabwe also support the idea that gender inequality and the lack of decision-making power of women resulted in women experiencing IPV [28, 29]. An innovative study by Pronyk et al. reported a reduction in IPV as a result of women being economically independent [38]. Therefore, eliminating gender inequalities and strengthening women’s economic and legal rights, needs to be encouraged and facilitated by governments [6].

Our study found the controlling behaviour of their partners had a debilitating and frustrating effect on women’s lives. The previous study conducted in Addis Ababa, Ethiopia, shows that partner controlling behaviour was one of the most prevalent types of violence [39]. It disconnected women from any possible support that they could obtain from neighbors, friends, relatives, and health care providers. It is also a violation of a person’s human right (as promulgated in the Ethiopian national revised family law in 2000 and the criminal law in 2005 [7, 9]), to be free from interference regarding one’s privacy, regarding one’s family, and home. The FGD discussants in this study suggested the need to enforce and teach both women and men together about human rights and IPV.

Women in this study believed that the legal service exists in Ethiopia to prevent violence, but that the implementation is weak, in that punishment on the ground is insufficient to teach the perpetrators a lesson to prevent them from continuing to engage in violent actions. Currently, however, this is a significant concern in many countries since the available law was not able to provide legal protection for women from violence in India, Sub-Saharan Africa, in the Middle East, nor in Ethiopia [9, 40]. Violence elimination is recognised as one of the international community’s priorities, and women have a right to live free from IPV [41]. In countries around the world (Brazil, Nepal, Spain, the United Kingdom, Uruguay, Venezuela, and several states in the USA), the government has set up special courts to deal expeditiously with these urgent cases since the health and lives of women are at stake [41, 42]. In Wolaita, however, women did not benefit from the legal services as they expected. Ethiopia needs a special court that deals with the IPV cases separately, and there is a need to decentralise the system as well as to train the legal bodies. The Sustainable Developing Goals (SDGs) emphasised the unacceptability of IPV by stating that all forms of discrimination and violence against women and girls will be eliminated (SDG, goal 5, article 5.2) [43]. The elimination process also proposed to engage men and boys, and Ethiopia can use this strategy in developing effective intervention programs for the prevention of violence against women.

Most of the women who were living both with and without HIV infection reported that educating women and men together about IPV is the best solution to prevent IPV. This finding accords with the World Development Report, which indicated that education should include both men and women and that the entire community should participate [40].

Finally, the new lessons learned from this study are the overlapping existence and nature of IPV and its prevention strategy. The prevention strategy proposed by women was that even when women knew their positive HIV status before their partners (in the case of very violent and aggressive partners), health care workers should test the women again as a new case along with their male partners and assist with the disclosure. Further, the results that emerged from this study highlighted the existence of weak and inappropriate punishment for perpetrators of violence, the absence of a women’s network working effectively on IPV, and that the existing groups of one leader to five women lacked an IPV prevention role. The women’s affairs office involvement in IPV was also found to be weak. However, the study found that the religious leaders’ role in IPV prevention is beneficial in Wolaita Zone.

### Strengths and limitation

The IPA design in this research allowed a detailed and in-depth description of Wolaita women’s IPV experiences and its meanings. Among other strengths, the unique perspective of the design provided a comprehensive understanding of the IPV phenomenon as experienced by the women, and the findings reported from the study result from the rich data and the open-ended structured guide that permitted the participants to talk freely so that the investigator gathered rich data for the analysis. Our research also provides evidence from multiple sources of the benefits from incorporating two methods of data collection namely, in-depth interviews and FGDs, and using data triangulation of the different sources of information (women on ART, family planning ANC/PMTCT users, etc.).We addressed the scientific rigor such as the credibility of the study to ensure that the data presented in the study reflects the views of the participants. During the data collection period, the team had peer debriefing, and the PI and the co-authors conducted the data analysis, and in the results section, we provided verbatim quotes. Moreover, we also offered transcripts to some participants to confirm their comments. To ensure transferability, we provided thick descriptions in the results section, to allow other researchers to decide whether the results are transferable to their context. The team conducted an audit trail with the supervisor and co-supervisor to ensure that the analysis is grounded in the data to maintain dependability. To ensure confirmability**,** this study triangulated data collection using in-depth interviews and FGDs.

However, our research has some limitations. Among these are the transferability and generalizability of these findings. Since we made every attempt to keep the descriptions faithful to the raw data, pure bracketing cannot be possible because the investigators interpreted the data based on their phenomenological world and experience. Hence, this process, in turn, may bring investigator-induced-bias to the study, but the researchers were aware of this and took note, and tried to avoid this possibility.

## Conclusion

The evidence from this study confirms that IPV is a considerable problem in Wolaita Zone and the extent of the different forms of violence (physical, sexual and psychological) which frequently overlapped, highlights the urgency of intervention measures. The five themes that emerged are: “women’s terrifying experiences of violence”, “the effects of violence on their health”, “support/lack of support /controlling behaviours/”, “women’s feelings about the available services”, and “IPV prevention strategy in the perspective of women”. Unwise disclosure and discordant results of HIV testing also exacerbated IPV. Women reported that the legal service exists but that the available punishment of perpetrators is insufficient and does not act as a deterrent. The study identified the lack of strong women’s networks, explicitly working on violence to resolve the conflict between partners and to punish the perpetrator. Among IPV prevention strategies, the participants mentioned that women should discuss the abuse they experienced with their neighbors, they should generate income in order not to be dependent on men, and they should teach their friends who lack awareness, how to obtain legal services. The law needs to be applied strictly and provide justice for all victims. Establishing women’s networks, which explicitly work on violence prevention, should be emphasized. Where women knew their serostatus before their husband/partner, they should be tested as a new patient together with their partners and disclosure should be assisted by health care providers. It would be essential to provide additional training for the health care providers giving counseling in such complex situations. Further investigation of the relationship between IPV and HIV should be examined in different parts of the country, to develop situation-specific approaches that may be needed.

## Supplementary information

**Additional file 1.** Interview guide (unstructured questionnaire).

**Additional file 2.** Socio demographic characteristics of the study participants.

**Additional file 3.** Code, category and themes.

**Additional file 4.** COREQ check list for qualitative study.

## Data Availability

The datasets generated and/or analyzed during the current study are not publicly available due to the sensitive nature of violence reported in this study, but are available from the corresponding author on reasonable request.

## Abbreviations

ART: Antiretroviral Therapy
EDHS: Ethiopian Demographic and Health Survey
FGD: Focus Group Discussion
IPV: Intimate Partner Violence
IPD: Interpretive Phenomenological Design
VAW: Violence Against Women
