# Supplementary material for: Lived experience of intimate partner violence among women using antiretroviral therapy and other outpatient services in Wolaita Zone, Ethiopia: a phenomenological study
Source: Reprod Health. 2021 Feb 1;18:25. doi: 10.1186/s12978-020-01044-0 (PMC7849132; doi:10.1186/s12978-020-01044-0)
Supplement: Supplementary file 1 — Additional file 1. Interview guide (unstructured questionnaire). [file 12978_2020_1044_MOESM1_ESM.docx]

**Appendices one: Information sheet, Consent and Questionnaire for Research (In-depth-Interview with Women)**

- 1. **Information Sheet and Consent to Participate in Research**

Date: ---------------------------------

Greetings, my name is -------------------------I provide the information about the researcher Mr. Mengistu Meskele Koyira, a Ph. D student at university of KuwaZulu -Natal, and former staff of Wolaita Sodo university, his contact address is (+251913177996 or E-mail address: mengistu77@gmail.com). You are being invited to consider participating in a study that involves research in **“Lived experience of intimate partner violence among women using antiretroviral therapy and other outpatient services in Wolaita Zone, Ethiopia: A phenomenological study ”**. The aim and purpose of this research is to learn about the women’s health and life experiences and to generate the local evidence. The study is expected to enroll up to 25 interviews but it depends on the information we get. We will conduct similar interviews at nine health facilities in Wolaita Zone. It will involve the following procedures. We are going to interview you, record the audio and take notes. The duration of your participation if you choose to enroll and remain in the study is expected to be 60 minutes.

The study may involve the following risks and/or discomforts. By participating in this research project, you may have minimum discomfort to disclose your painful experience, though, many women have found it helpful to have the opportunity to talk. we will also refer to psychological intervention or counselling service at health care facility in case you get discomfort. We (the research assistant) will also provide counselling at end of the interview. The study helps to generate local evidence to assist with the initiation and implementation of programs to address intimate partner violence against women.

This study has been ethically reviewed and approved by the UKZN Biomedical research Ethics Committee (approval number----------------------------------).

In the event of any problems or concerns/questions you may contact the researcher Mr.Mengistu Meskele (at +251913177996 or E-mail address: mengistu77@gmail.com) or the UKZN Biomedical Research Ethics Committee, contact details as follows:

**Biomedical Research Ethics Administration**

1. Research Office, Westville Campus
2. Govan Mbeki Building

Private Bag X 54001
Durban
4000

KwaZulu-Natal, SOUTH AFRICA

Tel: 27 31 2604769 - Fax: 27 31 2604609

Email: [BREC@ukzn.ac.za](mailto:ngwenyap@ukzn.ac.za)

Your participation in this research is completely voluntary. Your experiences could be very helpful to other women in Ethiopia. You may withdraw participation at any point, and that in the event of refusal/withdrawal of participation; the participants will not incur penalty or loss of treatment or other benefit to which they are normally entitled.

There is no direct benefit to your participation, but many women have found it helpful to have the opportunity to talk.

I kindly let you know that all of the answers you provide me will be kept secret. The record of your name or address will not be kept by the researcher. We will record the audios but all will have kept secret and your names will no longer be mentioned at any documents. Any information from the participants like raw data will be stored securely and kept confidential. The publications that will arise from this study will exclude any information that will make it possible to identify the participants’ name or identity. In case others enter the room, dummy questionnaires will be used.

- 1. **Consent**

I (-----------------------------------) have been informed about the study entitled (**Lived experience of intimate partner violence among women using antiretroviral therapy and other outpatient services in Wolaita Zone, Ethiopia: A phenomenological study** ) by (Mr. Mengistu Meskele Koyira).

I understand the purpose and procedures of the study.

I have been given an opportunity to answer questions about the study and have had answers to my satisfaction.

I declare that my participation in this study is entirely voluntary and that I may withdraw at any time without affecting any treatment or care that I would usually be entitled to.

I have been informed about any available compensation or medical treatment if injury occurs to me as a result of study-related procedures.

If I have any further questions/concerns or queries related to the study I understand that I may contact the researcher at (Mr. Mengistu Meskele (+251913177996 or E-mail address: mengistu77@gmail.com)

If I have any questions or concerns about my rights as a study participant, or if I am concerned about an aspect of the study or the researchers then I may contact:

**Biomedical Research Ethics Administration**

1. Research Office, Westville Campus
2. Govan Mbeki Building

Private Bag X 54001
 Durban
 4000

KwaZulu-Natal, SOUTH AFRICA

Tel: 27 31 2604769 - Fax: 27 31 2604609

Email: [BREC@ukzn.ac.za](mailto:ngwenyap@ukzn.ac.za)

Agree to audio recording Yes ------------ No-------------

Signature of Participant Date

____________________ _____________________

Witness name

1.----------------------------------- --------------------------------------

2.----------------------------------- --------------------------------------

Interviewer name

------------------------------------ ------------------------------------

- 1. **Questionnaires for In-depth-Interview with Women**

1. I would like a little about yourself? Have you attended formal school?
   - 1. Where is your residence now?
     2. Do you have children?
     3. How do you normally spend your days?
     4. What things do you like to do?
2. Tell me about your husband. How did you first meet? When did you get married?
   1. What does he do?
3. When did your problems with your husband start? How long has this continued?
   1. Are there times when this has improved, or gotten worse?
4. Can you give examples of physical violence in your community? In your life?

When and where does physical violence occur?

1. Are women forced to have sex with their partner/husband? Under what circumstances? Who usually decides how, when and how often to have sex between partners? Do men force their partners to have sex even if they do not want? Can you give examples of sexual violence in your community? In your life?

When and where does sexual violence occur?

1. Who are the perpetrators? For physical or sexual violence (PROBE: Your husband/ family member, people you know/don’t know.) What happens to the perpetrators?
2. What are the problems that women face after an attack? (PROBE: physical, psychological, social problems.)
3. How do survivors of physical violence cope after the attack? How do survivors of sexual violence cope after the attack?
4. What are community responses when sexual/physical violence occurs? What is done to prevent violence?
5. What is done to help survivors? How could these efforts be improved? Do women’s support networks exist to help survivors?
6. What social and legal services exist to help address these problems? (PROBE: health, police, legal counseling, social counseling.) Who provides these services? How could these efforts be improved?
7. Has the problem of physical/ sexual violence gotten worse, better, or stayed the same since you arrived in Wolaita?
8. Has it had a great effect on your physical well-being? In what ways?
9. How has it affected your feelings about yourself?
10. Do you think that it is having an effect on your children? In what ways?
11. Has it affected your ability to provide for the family or go to work?
12. Has it made it difficult for you to meet friends or relatives? How?
13. Have you ever discussed your problems with others? How did they respond?
    1. Was there more that you would have liked them to do?
    2. What sort of things would have helped?
14. Looking back at your situation, what advice would you give another woman
15. who has just started to have these sorts of problems with her husband?

**Questions for people living with HIV**

1. Do you think it is good for a woman to inform her partner about her HIV test results? Why do you say so? Probe.

22. Why do some women fail to collect their HIV test results?

a. Fail to join PMTCT/ART program? b. What makes some women decide to accept the test?

23. What is the best time/opportunity to inform the male partner?

24. How would you inform him if you wanted to?

25. How do you think a husband/partner might react after you tell him your status (that you are HIV positive)

26. Who else do you think women inform about their HIV status?

27. What do you think happens to a woman who makes her status known to her partner?

Probe several actions and behaviors!

28. What do you think happens when women tell other people about their status?

29. How involved are men in ART programme? Are men supportive of women to test

during pregnancy and other time?

**Thank you very much for your time!**

**Appendixes Two: Information sheet, Consent and Questionnaire for Research (Focused Group Discussion for women living with and without HIV)**

- 1. **Information Sheet and Consent to Participate in Research**

Date:---------------------------------

How are you? My name is ----------------------------- I provide the information about the researcher Mr. Mengistu Meskele Koyira, a Ph.D student at university of KuwaZulu -Natal, and former staff of Wolaita Sodo university, his contact address is (+251913177996 or E-mail address: mengistu77@gmail.com). You are being invited to consider participating in a study that involves research in **“Lived experience of intimate partner violence among women using antiretroviral therapy and other outpatient services in Wolaita Zone, Ethiopia: A phenomenological study ”**. The aim and purpose of this research is to learn about the women’s health and life experiences and to generate the local evidence. The study is expected to enroll up to four Focused Group Discussions but it depends on the information we get. We will conduct similar other discussions in the nine health facilities in Wolaita Zone. It will involve the following procedures. We are going to interview you, record the audio and take notes. The duration of your participation if you choose to enroll and remain in the study is expected to be 60 minutes.

The study may involve the following risks and/or discomforts. By participating in this research project, you may have minimum discomfort to disclose your painful experience, though, many women have found it helpful to have the opportunity to talk. we will also refer to psychological intervention or counselling service at health care facility in case you get discomfort. We (the research assistant) will also provide counselling at end of the interview. The study helps to generate local evidence to assist with the initiation and implementation of programs to address intimate partner violence against women.

This study has been ethically reviewed and approved by the UKZN Biomedical research Ethics Committee (approval number----------------------------------).

In the event of any problems or concerns/questions you may contact the researcher Mr.Mengistu Meskele (at +251913177996 or E-mail address: mengistu77@gmail.com) or the UKZN Biomedical Research Ethics Committee, contact details as follows:

**Biomedical Research Ethics Administration**

1. Research Office, Westville Campus
2. Govan Mbeki Building

Private Bag X 54001
Durban
4000

KwaZulu-Natal, SOUTH AFRICA

Tel: 27 31 2604769 - Fax: 27 31 2604609

Email: [BREC@ukzn.ac.za](mailto:ngwenyap@ukzn.ac.za)

Your participation in this research is completely voluntary. Your experiences could be very helpful to other women in Ethiopia. You may withdraw participation at any point, and that in the event of refusal/withdrawal of participation, the participants will not incur penalty or loss of treatment or other benefit to which they are normally entitled.

There is soft drinks like coca cola, tea and coffee for your time but no other direct benefit to your participation, but many women have found it helpful to have the opportunity to talk.

I kindly let you know that all of the answers you provide me will be kept secret. The record of your name or address will not be kept by the researcher. We will record the audios but all will have kept secret and your names will no longer be mentioned at any documents. Any information from the participants like raw data will be stored securely and kept confidential. The publications that will arise from this study will exclude any information that will make it possible to identify the participants’ name or identity. In case others enter the room, dummy questionnaires will be used.

- 1. **Consent**

I (-----------------------------------) have been informed about the study entitled (**Lived experience of intimate partner violence among women using antiretroviral therapy and other outpatient services in Wolaita Zone, Ethiopia: A phenomenological study** ) by (Mr.Mengistu Meskele Koyira).

I understand the purpose and procedures of the study.

I have been given an opportunity to answer questions about the study and have had answers to my satisfaction.

I declare that my participation in this study is entirely voluntary and that I may withdraw at any time without affecting any treatment or care that I would usually be entitled to.

I have been informed about any available compensation or medical treatment if injury occurs to me as a result of study-related procedures.

If I have any further questions/concerns or queries related to the study I understand that I may contact the researcher at (Mr. Mengistu Meskele (+251913177996 or E-mail address: mengistu77@gmail.com)

If I have any questions or concerns about my rights as a study participant, or if I am concerned about an aspect of the study or the researchers then I may contact:

**BIOMEDICAL RESEARCH ETHICS ADMINISTRATION**

1. Research Office, Westville Campus
2. Govan Mbeki Building

Private Bag X 54001
 Durban
 4000

KwaZulu-Natal, SOUTH AFRICA

Tel: 27 31 2604769 - Fax: 27 31 2604609

Email: [BREC@ukzn.ac.za](mailto:ngwenyap@ukzn.ac.za)

Agree to audio recording Yes ------------ No-------------

Signature of Participant Date

____________________ _____________________

Witness name

1.----------------------------------- --------------------------------------

2.----------------------------------- --------------------------------------

Interviewer name

------------------------------------ ------------------------------------

2.3. Questionnaire: FGD Guide for women living with and without HIV

1. What problems have women experienced in health in your community? **(PROBE** on violence, not on health.) What type of unwanted actions or behavior do men do to their partners? Probe financially, emotionally, physically, sexually? Probe
2. Can you give me the examples of physical violence in your community? In your life?

When and where does physical violence occur? Has it had a great effect on your physical well-being? In what ways?

1. How has it affected your feelings about yourself?
2. Are women forced to have sex with their partner/husband? Under what circumstances? Can you give examples of sexual violence in your community? In your life?

When and where does sexual violence occur? Who usually decides how, when and how often to have sex between partners? Do men force their partners to have sex even if they do not want? Are women abused for testing for HIV?

1. Who are the perpetrators? For physical/sexual violence **(PROBE**: Your husband/ family member, people you know/don’t know.) What happens to the perpetrators?
2. What are the problems that women face after an attack? (**PROBE**: physical, psychological, social problems.)
3. How do survivors of physical / sexual violence cope after the attack? Where do women get help (report) if they are abused by their partners?
4. Has it made it difficult for you to meet friends or relatives? How?
5. What are community responses when physical or sexual violence occurs? What is done to prevent violence?
6. What is done to help survivors? How could these efforts be improved? Do women’s support networks exist to help survivors?
7. What social and legal services exist to help address these problems? (PROBE: health, police, legal counseling, social counseling.) Who provides these services? How could these efforts be improved?
8. Has the problem of physical/ sexual violence gotten worse, better, or stayed?
9. Is there anything that religious leaders can do to prevent physical or sexual violence?
10. Is there anything women leaders can do to prevent sexual violence?

**Questions for people living with HIV**

1. Do you think it is good for a woman to inform her partner about her HIV test results? Why do you say so? Probe.

16. Why do some women fail to collect their HIV test results?

a. Fail to join PMTCT/ART program?

b. What makes some women decide to accept the test?

17. What is the best time/opportunity to inform the male partner?

18. How would you inform him if you wanted to?

19. How do you think a husband/partner might react after you tell him your status (that you are HIV positive)

20. Who else do you think women inform about their HIV status?

21. What do you think happens to a woman who makes her status known to her partner?

Probe several actions and behaviors! What do you think happens when women tell other people about their status?

22. What could possibly happen to a woman after she disclosed her HIV test results to a husband/ partner?

23. How involved are men in ART programme? Are men supportive of women to test during pregnancy and other time?
