# Supplementary material for: Lived experience of intimate partner violence among women using antiretroviral therapy and other outpatient services in Wolaita Zone, Ethiopia: a phenomenological study
Source: Reprod Health. 2021 Feb 1;18:25. doi: 10.1186/s12978-020-01044-0 (PMC7849132; doi:10.1186/s12978-020-01044-0)
Supplement: Supplementary file 3 — Additional file 3. Code, category and themes. [file 12978_2020_1044_MOESM3_ESM.docx]

Table 3: Themes emerged from the study

| Themes | **Women's terrifying**  **experiences of violence** | **The effect of violence on women's health** | **Support/lack of support / partner’s controlling behaviours)** | **Women’s feelings about the available service** | **IPV prevention strategy from the perspective of women** |
| --- | --- | --- | --- | --- | --- |
| Categories (Sub-themes) | 1-Physical violence  2-Emotional violence  3-Sexual violence  4-Violence reaction of partners after HIV result Disclosure  5-Cause for the terrifying abuse | 1-Women’s health  2-Children’s health  3- Social effect  4-Psychological effect  5-Economic effect /violence | 1-Partner controlling behaviour | 1-Legal service  2-Servises from community and religious leaders | -Right time to disclose HIV test result and its strategy to prevent IPV  - How women survive from IPV  -Women’s advice to others  - Solution and care for IPV victims  -Women’s network availability |
| Codes | **Physical violence**   - striking with a stick - Wife beating - chasing wife to bit - broke hand, hand fracture - lost one of my eyes and it became blind - He beat me intending to kill - killed their wives - kick me by his leg - broken my teeth - bleeding women - chest bone has broken - Quarrelling - He never abuses me physically - punched my face - My tooth changed their normal place - holding my hair - hair vanished in his hands - swelling - threw any material upon my face - He kicked her child; as a result the child died - Threw me on fire - threaten servants by pistil - hit her organ (vagina) - Lacerated her lips - kicked by his leg - stab with a knife - He wants to kill her - He wants to bleed her - he attempted to pierce me - **Emotional Violence** - hostile against me - He torched me psychologically. - I cried and preferred death. - I lost hope - Decided to drink poison and die - He is aggressive and people fear - threatened me to slaughter - threatening to kill - threatened to blind my eyes and insult - I want to enter into the lake and die - He was stigmatizing - Dropping a tear from my eyes - Always had fear - I was worried about his complain - He threatened by pistil - Demoralizing - Insult as she has multiple sexes - traumatized her psychologically, - he terrorized to kill by knife - He just argues - **Sexual Violence** - Practicing unfamiliar sex position which I didn’t want - Practiced sex in another organ left me a scar that I never forget - He forced me for sex which was painful - When we quarrel, he responds through sex - Presuming that she has an affair with someone else - He did the sex that I didn't want (practiced it anally) - Bleeding - My husband never forces me for sex - drunk and force me if he wants to have sex - If I refuse sex, he beats me and let to leave home - I feel tired but he wants to have sex daily - He has extramarital sexual partner - He didn't force me to have sex with him - used to rape servants - He sex immediately after delivery - He raped me - I had no right to have sex - No one other than husbands - feel pain in my uterovaginal area because of the sex - He doesn’t force me for sex - no cases of sexual violence in our community - He never forces me for sex(ANC) - My husband also does not force me for sex. - **Reactions of partner after HIV test result** - conflict on the issue - divorced me - marriage was affected - children will be disrupted - expel their wives from home - fearful of the community’s stigma - Refused to be tested - Restrict from social contact - Immediately can discriminate - Neighbor supplied coffee with a broken cup as a result of my HIV status - Restrict contact with friends - Discrimination is decreasing - In public, he stigmatizes me - stigmatizes me in front of all my parents - Insulting & chaos by inferring you have HIV - Insulted as you are baggers, and then I cried a lot - Our neighbors stigmatized me - Educated husband supports the test of HIV - disgusted with them and will talk about them - Husbands takeout their marriage rings - Good men accept it - Separate or divide every equipment they owned in common - Became angry - Some partners accept and comfort their wives | **Social effects**   - discriminated from the community - other community members discriminate her - feel loneliness - cannot freely engage in social life - Fear - can be demoralized by their friends - escaped to my parents (leave my home) - I developed a bad image upon the male - Restricting women’s right not to defend verbally   **Women health**   - Still, I have back pain - Abortion - I became lethargic; exhausted and about to die - Sequel of pain around my ear - I have been bleeding - Physical disability - In ability to work - Divorce - HIV/STI acquisition - Utrro-vaginal prolapse, - uterine infections and others - I don't like to watch TV, and noise, etc. - Depression - Always eyes are tearing - In ability to marry again - Abortion of twin pregnancy - Wanted suicide - Inability to control urine - Death of women - divorced from him - women were traumatized psychologically - bleeding after he had beat - I can’t engage in physically demanding work because of the pain - I acquired HIV from my second husband. - I dropped education at 7th grade prior to my first marriage - I already became pregnant. - Yes, it brought me HIV illness. - **Child health** - Child fall and death - Her child was died, - divorced her - while we fighting each other , our kids get hurt (injury of children) - I myself and my kids were affected. - **Economic Consequence** - I got nothing during our divorce(Preventing property sharing) - He was hiding many things - Men also don’t give their wives enough money - Provide nothing to his children from his income (No support to children) - Suffering me financially during all my life - Bankrupted his own money - Do not give money for food - No share of property - Restricting job - Hurt in administrative issues - burn my parent’s house - I left him and came back here empty-handed. | **Controlled behavior**   - He closed the door up on me and I was alone - Restricted my freedom - he protected me not to go outside the home - Restricted to speak only through the window - Abducted and took me another country where I have no support - Restricted speech with him (If I respond a single word to him, he started to beat me) - Restricted contact with friends (no friends were allowed to ask me) - Loneliness (He used to lock the door upon me) - I couldn’t make call to contact my friends - Restricted phone call - He was restricting my movement - Repeated name calling - threatened me as he already knows the place I was a - Suspicious that I was not faithful (suspicious as I having sex with other men ) - Restrict contact with friend - Protect access of other people who wanted to help me - Restrict sharing their thought - he prevents me from getting out of the house - Restrict contact with our neighborhood to talk - talk extra words - tell them to get out of their house - denied his own child - he used to chase our children - shifted his anger - He always disturbs me - He left me home | **Legal services**   - It is not that strong - There is no legal service to go - Lack of awareness to use legal support - There is women's affairs organization - response from legal bodies were not satisfactory - For men who deserve punishment, women's affair didn't punish - women's affair reluctant to punish - No appropriate punishment - Husbands taken to court - The religious leaders usually have a concern on the issue of violence - Religions leaders have a good role and sacrifices their time to resolve - The religious bodies are helpful - Referral to law - Now there is the legal rule - Husbands were forcing the young girl to report and lie as if they are their wife - Releasing the perpetrators from jail freely or very soon - Husbands use their money to free them from charge - The jury decide few money to care children - There are legal response to the violence act - Women cannot accuse their husband - Strong women who able to accuse her husband encounter discrimination from the community - Community terrify strong women - the women fears to accuse - There is politics about it in theory but there is no support. - Husbands restrict contact with strong women - It affects my feeling - stigmatized her as she accused her husband; - will be released after two days through guarantee - The court referring women to community elders or church elders after long follow up and appointment - A bit difficult to reach the religious leaders - Only punished the perpetrator in prison for three to four days. - justice is nothing, it is loose - the perpetrator could not pass a night in jail - The punishment is loose. | **Right to Disclose HIV result**   - Health professional test partners together, counsel and tell their HIV status when the couples are together - In case of discordant result, women should be tested again as a new client together with her husband in order to convince him and disclosure assisted by health care provider to prevent IPV - Women living with HIV should share their experiences to others - Disclosure should be made when women become pregnant - Disclose should be made in husbands appropriate time, when they are comfortable, and after dinner - when partners drink coffee together or at night - Disclosing HIV test made in the strategic test in mass campaign in their district, - Disclosure made when women sick, time of marriage - When both are at home in private   before going to bed   - tell them in ART class together with the ART focal   **survival strategy**   - Women should leave all their children in his home or take all her children along with her and leave his home. - Policeman or elders of the neighbor can resolve her issue. - Accepting husband very politely while he was drunk - Become wise, polite and humble in front of the husband - Divorce is good strategy - Referred her to women's and child affairs   **Women’s advice to others**   - to manage their home to generate source of income by working - It is better if the women advise their husbands wisely - Awareness creation - women who were already affected by abuse should teach and learn each other in an organized manner - women to report the abuse to government - better for women if the law is very strict and provides justice - I left my home and flee - **Solutions and care for the victims** - they will get first aid - they undergo a medical check-up - they should referred to the district or loca women's affair offices - woman and her family should follow the case strictly otherwise they will not get the right judgment - Perpetrators should be punished appropriately - Establishing an organization working on violence - I left my home and flee - Nothing is done for me. - There is no one who addresses the problems. - The religious bodies are helpful - our relatives settled our issue - Awareness creation. - women association is good by itself - Gather men and teach in a group in order to prevent their abusive behaviours - women and men get education together in violence issue - After the attack women should consult their friends, go to court, consult their children, reassure them, and convince themselves - The 1 to 5 organization should work strongly - Elders can fix our problems - Report such cases to the police - Take the case to court or they send elders to solve the dispute - Legal services exist - Discussing their case with others - A woman should work so hard to be respected at her home - **Women network availability** - There is no women network specifically working on this issue - There is only a women's affair office. - Female groups, like 1 to 5 leaders have no good role now - There are no strong and organized women leaders - Women's leader is not function but they are in place - So women’s affairs are a symbol. - There is a 1 to 5 and   1 to 30 network |
