# Supplementary material for: Lived experience of intimate partner violence among women using antiretroviral therapy and other outpatient services in Wolaita Zone, Ethiopia: a phenomenological study
Source: Reprod Health. 2021 Feb 1;18:25. doi: 10.1186/s12978-020-01044-0 (PMC7849132; doi:10.1186/s12978-020-01044-0)
Supplement: Supplementary file 4 — Additional file 4. COREQ check list for qualitative study. [file 12978_2020_1044_MOESM4_ESM.docx]

Table 1. Socio demographic characteristics of the in depth-interview participant women who were living with and without HIV in Wolaita Zone, Ethiopia, November 2018 -December 2018

| S.N. | Participants’ Code | Age | Marital Status | Residence | Education | Occupation | Sero-Status |
| --- | --- | --- | --- | --- | --- | --- | --- |
|  | B#1 | 35 | Divorced | Urban | 10+4 | Employed | HIV+ |
|  | O#2 | 32 | Divorced | Urban | 8 | Employed | HIV+ |
|  | B#3 | 35 | Married | Urban | 3 | Housewife | HIV- |
|  | G#4 | 35 | Married | Urban | 12+1 | Employed | HIV- |
|  | D#5ANC | 28 | Married | Rural | 9 | Employed | HIV- |
|  | B#6 | 27 | Married | Urban | 12+3 | Employed | HIV- |
|  | G#7 | 28 | Widowed | Urban | 10 | Employed | HIV+ |
|  | O#8 | 35 | Married | Urban | 10 | House wife | HIV+ |
|  | B#9 | 38 | Divorced | Urban | 12 | Merchant | HIV- |
|  | D#10 | 30 | Married | Urban | 7 | Employed | HIV- |
|  | Bale#11 | 45 | Widowed | Urban | 5 | Housewife | HIV+ |
|  | O#12 | 35 | Widowed | Urban | 9 | Merchant | HIV+ |
|  | T#17 | 40 | Married | Urban | 3 | Merchant | HIV+ |

Table 2. Socio demographic characteristics of FGD participant women who were living with and without HIV in Wolaita Zone, Ethiopia, November 2018-December 2018

| Code | Age | Education | Occupation | Residence | Marital Status | Seo-status |
| --- | --- | --- | --- | --- | --- | --- |
| S2#1 | 32 | 4 | Employed | Urban | Widowed | HIV+ |
| S2#2 | 35 | 4 | Housewife | Urban | Married | HIV+ |
| S2#3 | 40 | Not-educated | Housewife | Urban | Widowed | HIV+ |
| S2#4 | 40 | Not-educated | Housewife | Urban | Widowed | HIV+ |
| S2#5 | 40 | 2 | Housewife | Urban | Widowed | HIV+ |
| S2#6 | 30 | Not-educated | Trader | Urban | Separated | HIV+ |
| S2#7 | 25 | Not-educated | Trader | Urban | Married | HIV+ |
| S2#8 | 28 | Not-educated | Trader | Urban | Married | HIV+ |
| S2#9 | 40 | 9 | Employed | Urban | Married | HIV+ |
| S1#1 | 38 | 8 | House Wife | Urban | Widowed | HIV+ |
| S1#2 | 25 | 8 | Trader | Urban | Widowed | HIV+ |
| S1#3 | 38 | 8 | House wife | Urban | Separated | HIV+ |
| S1#4 | 30 | 5 | House Wife | Urban | Married | HIV+ |
| S1#4 | 30 | 10 | Trader | Urban | Widowed | HIV+ |
| S1#5 | 29 | 9 | Volunteer | Urban | Widowed | HIV+ |
| S1#6 | 37 | 9 | Volunteer | Urban | Widowed | HIV+ |
| S1#7 | 38 | 10 | Volunteer | Urban | Separated | HIV+ |
| S1#8 | 30 | 9 | Volunteer | Urban | Married | HIV+ |
| S1#9 | 40 | 10 | Adherence counselor | Urban | Divorce | Sero-status |
| T#1. | 32 | 9 | Trader | Urban | Married | HIV+ |
| T#2. | 28 | 10 | Employer | Urban | Married | HIV+ |
| T#3. | 25 | 12+3 | House Wife | Urban | Divorced | HIV+ |
| T#4. | 30 | 8 | Trader | Urban | Married | HIV+ |
| T#5. | 30 | Not-educated | Trader | Rural | Married | HIV+ |
| T#6. | 40 | Not-educated | House Wife | Urban | Divorced | HIV+ |
| T#7. | 30 | Not-educated | House Wife | Rural | Married | HIV+ |
| D#1. | 23 | 10+4 | Employed | Urban | Married | HIV- |
| D#2. | 20 | 8 | Trader | Rural | Married | HIV- |
| D#3. | 21 | 7 | Trader | Urban | Married | HIV- |
| D#4. | 20 | 6 | House Wife | Urban | Married | HIV- |
| D#5. | 23 | 7 | Trader | Urban | Married | HIV- |
| D#6. | 33 | 7 | House Wife | Urban | Married | HIV- |
| D#7. | 25 | 10+4 | Employed | Urban | Married | HIV- |
